# Exploring the association between asthma and chronic comorbidities: impact on clinical outcomes

**DOI:** 10.3389/fmed.2024.1305638

**Published:** 2024-01-26

**Authors:** Aditya Sri Listyoko, Ryota Okazaki, Tomoya Harada, Genki Inui, Akira Yamasaki

**Affiliations:** ^1^Division of Respiratory Medicine and Rheumatology, Department of Multidisciplinary Internal Medicine, Faculty of Medicine, Tottori University, Yonago, Japan; ^2^Pulmonology and Respiratory Medicine Department, Faculty of Medicine, Brawijaya University-Dr. Saiful Anwar General Hospital, Malang, Indonesia

**Keywords:** ACO, asthma, comorbidity, mortality, outcomes

## Abstract

Asthma remains a significant global health challenge. While both the incidence and mortality rates have shown a decline, older individuals with asthma exhibit not just more severe symptoms but also demonstrate an elevated mortality rate. This phenomenon could be attributed to the presence of chronic comorbidities that exert an influence on clinical outcomes among adult patients with asthma. This review aims to present various aspects of asthma comprehensively, including the prevalence, incidence, mortality rates, and causes of death in adult patients with asthma. Additionally, this review delves into the impact of chronic comorbidities that contribute to the morbidity and mortality of patients with asthma on a global scale, encompassing conditions such as chronic kidney disease, diabetes mellitus, lung cancer, obesity, and cardiovascular disease, concerning asthma. Furthermore, the manuscript reviews the distinctions between asthma and asthma chronic obstructive pulmonary disease overlap and adds perspective on asthma as an occupational lung disease. Thus, this review aims to enhance clinicians’ awareness of the significance of chronic comorbidities in the management of patients with asthma. It seeks to provide insights that contribute to a more comprehensive approach to managing patients with asthma who also have comorbid conditions.

## 1 Introduction

Asthma is a chronic inflammatory disease of the airways with variable expiratory airflow limitation and diverse respiratory symptoms, remains a prevalent global health concern. Its spectrum of symptoms includes coughing, chest tightness, wheezing, and breathlessness, often triggered by an array of environmental factors, such as allergens, pollution, infections, weather changes, and emotional stressors (1). The primary objective of asthma management is to achieve well-controlled asthma based on symptom score, mitigate the risk of exacerbations and mortality, minimize the treatment side effects, particularly high-dose inhaled corticosteroids or oral corticosteroids, as well as preventing the onset of persistent airflow limitation that may lead to poor clinical outcomes (1, 2).

Numerous factors could have an impact on the clinical course of asthma and its management. Inadequate control of asthma (3, 4), short-acting β-agonist overuse (5, 6), improper use of inhaler devices (7), a history of severe exacerbations (8–10), and intrinsic conditions, such as reduced lung function and elevated blood eosinophil levels (11, 12) are among the factors that heighten the risk of future exacerbations and worsen clinical outcomes. Comorbidities such as higher body mass index (BMI) and obesity (13–15), obstructive sleep apnea (16, 17), food allergy (18, 19), allergic rhinitis (20), chronic rhinosinusitis with or without nasal polyposis (15, 21–23), psychological problems (21, 24), gastroesophageal reflux disease (GERD) (15, 25, 26), and also exert a significant influence on asthma-related outcomes. Concurrently, the presence of chronic diseases requiring regular medical treatment and evaluation, such as chronic kidney disease (CKD), diabetes mellitus (DM), lung cancer, obesity, cardiovascular disease (CVD), and asthma–chronic obstructive pulmonary disease (COPD) overlap (ACO) may impact asthma-related management, patients’ prognosis, quality of life, and contribute to asthma-related outcomes.

Chronic airway inflammation, characterized by variable expiratory airflow limitation, is a hallmark of the pathophysiological condition in asthma. Various substances, including allergens, viruses, bacteria, fungi, pollutants, and other harmful agents, can impact the epithelial airway—serving as the initial line of defense—initiating a cascade of inflammation (27). This cascade begins with the production of alarmins, including thymic stromal lymphopoietin (TSLP), IL-25, and IL-33 (28–30). In the subsequent phase, antigen-presenting cells (APCs) induce the differentiation of naïve T-cells into Th2 cells. Additionally, Th2 cells and innate lymphoid cells-2 (ILC-2) play a role in inducing eosinophilic inflammation through the release of IL-5 and IL-13 (31, 32). Eosinophilic products such as eosinophil cationic protein (ECP), eosinophil peroxidase (EPO), eosinophil-derived neurotoxin (EDN), and major basic protein (MBP) prove detrimental to the airways, triggering a cascade of inflammation, and numerous studies have observed an increase in the bronchial and serum levels of asthmatic patients (33–38). Th2 cells, via IL-4 and IL-33, induce the production of IgE by B-cells, which binds to mast cells, prompting their degranulation and releasing products such as prostaglandin-2 (PGD2), leukotrienes, and histamine (39–42). On the other hand, the differentiation of naïve T-cells into Th1 and Th17 primarily induces neutrophilic inflammation, producing non-Th2 cytokines like IL-6, IL-8, IL-17, IFN-γ, and TNF-α (43–46). This, in turn, triggers the production of neutrophilic products such as neutrophil extracellular traps (NETs), neutrophil elastase (NE), and myeloperoxidase (MPO) (47–50). Emerging evidence underscores the role of systemic inflammation, marked by elevated systemic inflammatory markers, in a subgroup of patients with asthma (51, 52). Systemic inflammation is more pronounced in neutrophilic inflammation, as evidenced by increased systemic inflammation markers such as CRP and IL-6 when compared to non-neutrophilic asthma (53). This systemic inflammation, if sustained, might foster the development of asthma-related comorbidities. However, the underlying mechanisms remain elusive, necessitating both laboratory and clinical research to unravel the intricate relationship between asthma and its associated chronic comorbidities.

Clinicians must recognize that achieving the goals of asthma management in patients who have concurrent chronic medical conditions necessitates a comprehensive approach that extends beyond solely addressing asthma-related treatment strategies. In these cases, the clinical focus should encompass effective management of both the primary asthma condition and the associated comorbidities, which can collectively impact disease progression, symptom severity, and overall patient well-being. This integrated approach acknowledges the intricate interplay between asthma and chronic comorbidities, thereby paving the way for more comprehensive and effective patient management strategies. Here, we review various aspects of asthma, particularly intending to understand the impact of various comorbidities on patients with asthma, to provide clinicians with insights that may facilitate an integrated approach to the management of asthma in patients with comorbid conditions.

## 2 Asthma prevalence, incidence, and mortality rate

Asthma remains a major global health challenge, affecting a substantial number of people globally. According to a systematic analysis of 220 population-based studies, the estimated prevalence of asthma cases and related symptoms (evaluated by reported symptoms), including reported wheezing (current and history of wheezing) was 754.6 million (95% confidence interval [CI]: 599.7–943.4), 1181.3 million (95%CI: 938.0–1471.0), respectively where current asthma and history of asthma was 357.4 million (95%CI: 213.0–590.8), 645.2 million (95%CI: 513.1–806.2), respectively (54). Concurrently, the Global Burden of Disease study in 2019 reported a positive shift with declining asthma incidence, reducing from 601.20 per 100,000 to 477.92 per 100,000 between 1990 and 2019, along with decreased asthma mortality, down from 8.60 per 100,000 to 5.96 per 100,000. However, the study also underscored age-specific vulnerabilities, with children exhibiting a high relative risk (RR) of incidence, whereas older individuals had a heightened relative risk of mortality (55).

Although the incidence of asthma appears to be declining, asthma’s enduring impact persists, particularly among older individuals, who experience more severe manifestations and higher mortality rates than children. Furthermore, an alarming observation revealed that children and young adults with asthma continue to encounter a heightened risk of mortality, regardless of underlying conditions that limit life expectancy or the socioeconomic status of parents, with an adjusted hazard rate of 1.46 (95%CI: 1.33–1.62) for all-cause mortality (56). Additional evidence from cohort studies unveiled a standardized mortality ratio (SMR) of 1.24 (95%CI: 1.11–1.37, *p* < 0.001) for all-cause mortality in adult patients with asthma (aged ≥15 years) (5). Furthermore, the impact of asthma extends beyond mortality, imposing a substantial disability burden: it ranks 23rd among diseases when considering its impact on disability, as measured by disability-adjusted life years (DALYs) (57).

## 3 Cause of death in adult asthma patients

The specific cause of death among patients with asthma (Table 1) ranges widely and includes malignant diseases including lung cancer, benign conditions including COPD, CVD, respiratory tract infections (pneumonia and influenza), as well as other causes such as diseases of the musculoskeletal system and connective tissue, diseases of the digestive system, diseases of the genitourinary system, endocrine and metabolic diseases and immunity disorders, diseases of the skin and subcutaneous tissue, external causes of injury and poisoning, complication of pregnancy and other diseases (58–63). Through a retrospective analysis, asthma was identified as a notable risk factor for increased all-cause mortality (56, 64). Among patients with asthma, malignant disease has become the primary cause of death in individuals with asthma, with pneumonia and cardiovascular disease following closely behind in terms of mortality. Within the category of malignant diseases, lung cancer exhibited the highest mortality rate, followed by hepatobiliary, gastrointestinal, and gynecological cancers (60). In another study, the elevated mortality risk among adults with asthma was predominantly attributed to the emergence of COPD and lung cancer, with hazard ratios (HR) of 12.0 (95%CI: 4.18–34.2, *p* < 0.001) and 2.33 (95%CI: 1.25–4.42, *p* = 0.008), respectively (61).

**Table 1:** Specific causes of death in asthma patients.

References	Research type	Sample size and group		Cause of death	HR
		Study group	Control group		
Huovinen et al. (58)	Population-based cohort study	64 asthma patients	1,693 non-asthma participants	COPD Lung cancer	2.09 (0.72–6.02) 2.36 (0.88–6.34)
Soto-Campos et al. (59)	Retrospective case–control study	82 asthma patients	2,461 non-asthma/COPD participants	Solid malignancy CVD (most commonly heart failure)	n/a n/a
Yamasaki et al. (60)	Retrospective study	54 asthma patients	n/a	Malignant disease (most commonly caused by lung cancer) Benign disease (most commonly caused by pneumonia)	n/a n/a
Lemmetyinen et al. (61)	Population-based cohort study	221 asthma patients	335 non-asthma patients	Neoplasm of respiratory organ COPD	2.35 (1.25–4.42) 12.0 (4.18–34.2)
Strand et al. (62)	Cohort study	3,314 active asthma patients	431,429 non-asthma participants	CVD (current asthma) CHD (current asthma) CVA (current asthma)	1.32 (1.08–1.62) 1.16 (0.78–1.73) 1.23 (0.86–1.74)
He et al. (63)	Population-based prospective cohort study	394 current asthma and 121 former asthma patients	3,811 non-asthma participants	CVD (current asthma) Heart disease (current asthma) Chronic lower respiratory disease (current asthma)	1.41 (1.08–1.85) 1.46 (1.06–2.00) 4.67 (2.84–7.70)

COPD, chronic obstructive pulmonary disease; CVD, cardiovascular disease; CVA, cerebrovascular accident; CHD, coronary heart disease; n/a, not available.

Interestingly, a prospective cohort study conducted among a population revealed that ongoing asthma raised the risk of overall mortality, chronic lower respiratory disease, and CVD mortality in adult asthma patients, and this trend was not observed in individuals with a history of asthma (63). In addition, this study identified ongoing asthma as a significant risk factor for higher mortality in patients with chronic lower respiratory system diseases (HR 3.17; 95%CI: 1.96–5.14) and found an association between current asthma and an increased risk of CVD-related mortality (HR 1.41; 95%CI: 1.08–1.85) (63). Furthermore, asthma exacerbations were shown to contribute to asthma-related mortality, with data analysis from the Nationwide Inpatient Sample (NIS) revealing an overall hospital mortality rate of 0.5% in patients aged >5 years of age (65).

## 4 Comorbidities act as factors that increase the risk of asthma exacerbation

“Comorbidity” refers to the coexistence of additional diseases alongside the index disease within the same individual, while multimorbidity denotes the presence of multiple diseases within a single individual (66). The assessment of comorbidities holds significant relevance to the management of asthma. Certain comorbidities have been firmly established as asthma exacerbation risk factors. Furthermore, the investigation of comorbidities becomes integral to the difficult-to-treat and severe asthma management, wherein patients continue to experience exacerbations and suboptimal symptom control despite receiving optimal treatment (1).

**Figure 1:**
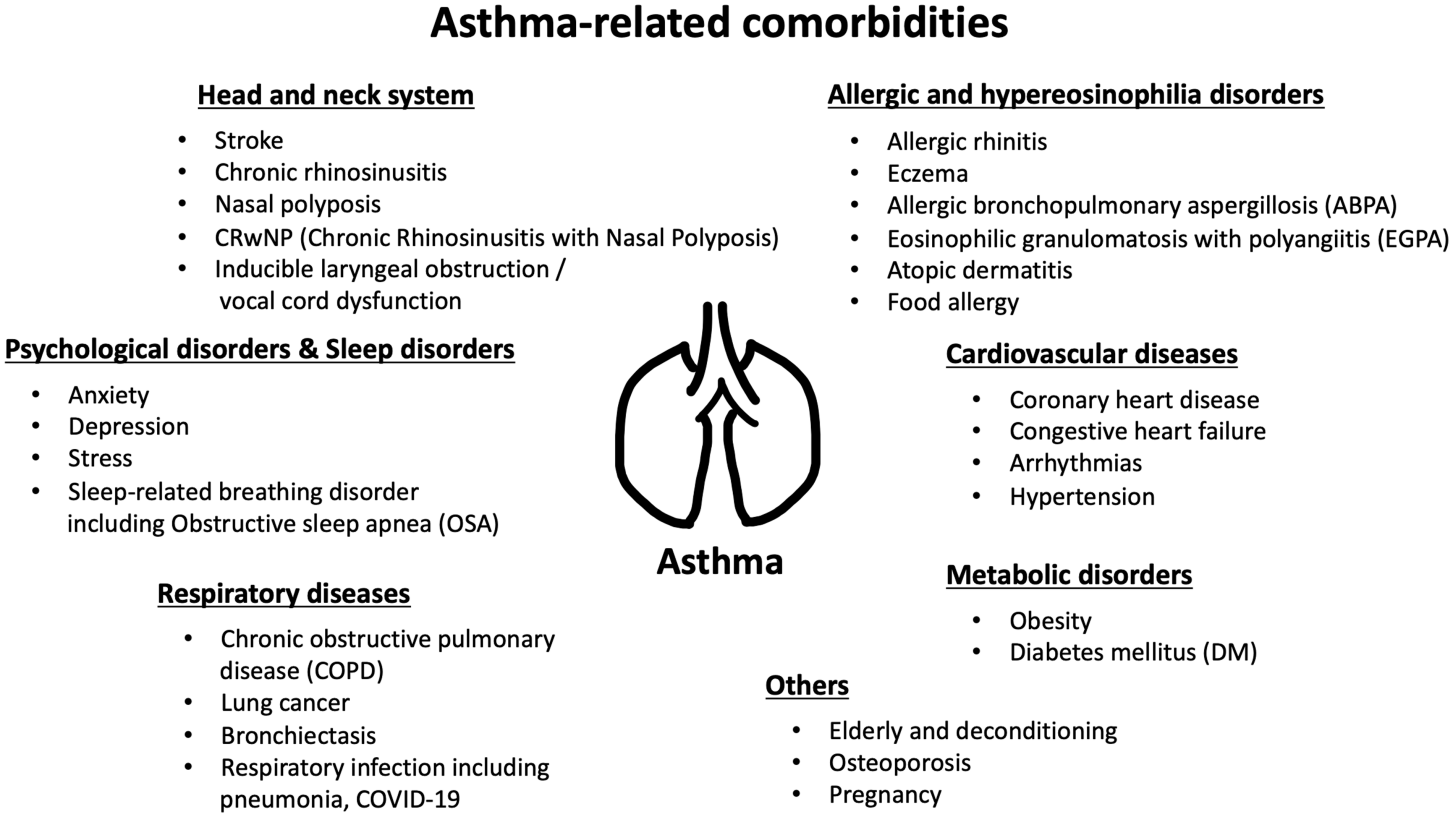
Asthma-related comorbidities have been reported in guidelines or literature.

Asthma-related comorbidities can manifest in diverse systems and organs throughout the body. This broad spectrum of associated conditions underscores the systemic nature of asthma. It goes beyond being just a chronic inflammatory airway disease, extending into a systemic immunological condition, with the potential to impact overall health. These comorbidities can range from cardiovascular issues and metabolic disorders to respiratory complications and psychological health challenges (Figure 1). Numerous comorbidities have been thoroughly evaluated and identified as significant factors that increase the risk of exacerbations. For instance, a prospective study discovered a correlation between obesity and a heightened likelihood of occurrences such as short-acting beta-agonist (SABA) canister dispensation, oral corticosteroid (OCS) dispensation, and emergency room (ER) visits or hospitalizations (67). Individuals with obesity and asthma exhibit heightened exacerbation severity, as evidenced by an elevated risk of requiring noninvasive positive-pressure ventilation and mechanical ventilator support (68). Other established comorbidities that elevate exacerbation risk include chronic sinusitis and psychological disorders. Psychological disorders, including depression, anxiety, as well as severe chronic sinus disease, have been evaluated as independent factors contributing to frequent exacerbations (21, 69, 70).

GERD, food allergies, and pregnancy are also recognized as having notable impacts on future asthma exacerbations. Patients with asthma with coexisting GERD often experience worse symptom control than those without GERD, which in turn is associated with exacerbation frequency and the need for OCS treatment (25, 26). An insightful cross-sectional study highlighted that patients with multiple food allergies face a higher likelihood of asthma-related hospitalizations, ER visits, and oral steroid use (71). Pregnant women with asthma are likewise susceptible to exacerbations, with a prospective study revealing that 36% of pregnant subjects with asthma experienced severe exacerbations, particularly those with severe asthma (72). In this population, having a history of exacerbations and using medium to high doses of inhaled corticosteroids (ICS) are identified as risk factors for severe exacerbations (73).

Importantly, chronic conditions may evolve and contribute to the emergence of new asthma-related comorbidities, subsequently influencing clinical outcomes, including exacerbations. Addressing these comorbidities as part of asthma management is crucial for achieving well-controlled asthma and minimizing the risk of future exacerbations.

## 5 The impact of chronic comorbidities on asthma clinical outcomes

Chronic comorbidities exert a profound influence on the global disease burden. These conditions, characterized by their persistence and concurrent presence with primary diseases, have garnered increasing attention due to their substantial impact on public health, healthcare systems, and the overall well-being of individuals. As populations age and lifestyles evolve, the prevalence of chronic comorbidities continues to rise, presenting multifaceted challenges to healthcare providers. Asthma, in addition to being a chronic respiratory condition in its own right, is closely associated with several chronic comorbidities that can significantly impact its clinical outcomes. Below, we delve into a spectrum of common chronic comorbidities that exert a significant influence on both morbidity and mortality within the general population and highlight their relationship to asthma and its clinical outcomes (Table 2).

**Table 2:** The relationship between asthma and chronic comorbidities and the impact on clinical outcome.

Comorbidities	References	Research type	Sample size and group		Outcome	Unmet need
			Study group	Control group		
CKD	Huang et al. (74)	Retrospective cohort	35,086 asthma subjects	105,258 non-asthma subjects	Asthma ↑ CKD development (HR 1.56, 95%CI: 1.48–1.64)	The impact of CKD or renal impairment on asthma outcomes, including exacerbation, hospitalization, and mortality, and on the success of biologic agents treatment in severe asthma
	Adawy et al. (75)	Cross sectional	118 asthma subjects	118 healthy subjects	Asthma ↑ CKD development vs. healthy subject (17.4% vs. 0.8%) Positive correlation between GFR and %FVC; %FEV1; FEV1/FVC, %PEFR	
DM	Ehrlich et al. (76)	Retrospective cohort	70,645 DM subjects	51,241 non-DM subjects	DM ↑ asthma development (HR 1.08, 95%CI: 1.03–1.12)	The impact of DM on the biologic agents administration in severe asthma patients
	Hsiao et al. (77)	Retrospective cohort	3,545 type-1 DM subjects	14,180 non-DM subjects	DM ↑ asthma development (HR 1.34, 95%CI: 1.11–1.62) DM ↑ asthma exacerbation (ER visit) (HR 17.4, 95%CI: 12.9–23.6) DM ↑ asthma hospitalization (HR 38.6, 95%CI: 28.5–52.2)	
	Wu et al. (78)	Retrospective cohort	2,716 and 934 pre-diabetes and DM subjects	2,072 normal HbA1C subjects	Pre-diabetes ↑ asthma exacerbation (not specified: hospitalization, ER visit, OCS prescription) (HR 1.27, 95%CI: 1.05–1.52) DM ↑ asthma exacerbation (not specified: hospitalization, ER visit, corticosteroid prescription) (HR 1.33, 95%CI: 1.02–1.73)	
	Lee et al. (79)	Retrospective study	24 asthma-DM subjects	134 asthma-non-DM subjects	DM ↑ asthma development (HR 1.75, 95%CI: 1.06–3.02); HbA1C ↑ asthma development (HR 1,38, 95%CI: 1.03–1.84)	
	Engelkes et al. (80)	Retrospective cohort	Asthma-DM 5,001 548 2,244 20,317 4,901	Total asthma 73,506 14,041 37,003 393,660 68,226	DM ↑ mortality of asthma patients in 5 years HR 2.14, 95%CI: 1.82–2.52 HR 1.57, 95%CI: 1.21–2.05 HR 1.18, 95%CI: 0.99–1.41 HR 1.70, 95%CI: 1.61–1.80 HR 1.66, 95%CI: 1.50–1.84 Metaanalysis result: HR 1.61, 95%CI: 1.33–1.95	
	Yang et al. (81)	Cross sectional	2,320 asthma with pre-DM/DM	45,286 asthma with normal HbA1C	HbA1C level (pre-DM/DM) ↑ asthma-related hospitalization (HR 1.68, 95%CI: 1.18–2.41) HbA1c was significantly and inversely associated with FEV1 and FVC	
Lung cancer	Huovinen et al. (58)	Population-based cohort	11 deaths asthma+lung cancer subjects	104 non-asthma+lung cancer subjects	Asthma ↑ lung cancer mortality (HR 2.36, 95%CI: 0.88–6.34)	The impact of lung cancer on asthma outcomes, including exacerbation, hospitalization, and mortality, and on the success of biologic agents treatment in severe asthma
	Boffetta et al. (82)	Population-based cohort	713 lung cancer among asthma subjects	92,986 hospital-discharge asthma subjects	Asthma ↑ lung cancer, incidence ratio 1.58 (95%CI: 1.47–1.70)	
	Pelkonen et al. (83)	Cross-sectional	9 hospitalized-asthma + lung cancer subjects	80 hospitalized non-asthma + lung cancer subjects	↑ mean hospital days/year because of lung cancer (asthma vs. non-asthma group: 0.14 vs. 0.03)	
	Kantor et al. (84)	Prospective cohort	8,442 asthma+lung cancer subjects	102 non-astma+lung cancer subjects	Asthma ↑ lung cancer risk (HR 1.25, 95%CI: 1.00–1.57)	
Obesity	Beuther et al. (85)	Metaanalysis prospective study	333,102 subjects who have asthma incident	n/a	Overweight and obesity (BMI ⩾ 25) ↑ asthma incident (OR 1.51, 95%CI: 1.27–1.80)	The impact of obesity on airway remodeling and on the success of biologic agents treatment in severe asthma.
	Huang et al. (86)	Case control	100 obese poorly-controlled asthma	225 obese well-controlled asthma	Obesity was significantly associated with poorly controlled asthma (OR 1.49; 95%CI: 1.09–2.03)	
	Rodrigo et al. (87)	Prospective cohort	161 asthma patients with BMI ≥ 25	265 asthma patients with BMI < 25	Patients with BMI ≥ 25 kg/m2 ↑ length of ED stay (2.3 h vs. 1.9 h) and rate of hospitalization (13.7% vs. 6.8%)	
	Sturesson et al. (88)	Population-based cohort	328 obese asthma subjects	940 normal-weight asthma subjects	Obesity ↑ all-cause mortality (HR 1.26, 95%CI: 1.03–1.54) and cardiovascular mortality (HR 1.43, 95%CI: 1.03–1.97).	
HT	Ferguson et al. (89)	Cross-sectional	191 asthma+HT subjects	621 asthma+non-HT subjects	HT ↓ FEV1(%): 70–79% (OR 1.47, 95%CI: 0.82–2.64); 60–69% (OR 2.53, 95%CI: 1.18–5.46); <60% (OR 1.01, 95%CI: 0.45–2.29)	The impact of HT on the success of biologic agents treatment in severe asthma.
	Lee et al. (79)	Retrospective cohort	58 asthma+HT subjects	100 asthma+non-HT subjects	HT ↑ asthma development (HR 1.43, 95%CI: 1.09–2.10)	
	Lin et al. (90)	Retrospective study	652 severe-life threatening exacerbation subjects	2,557 mild–moderate exacerbation subjects	HT ↑ severity of exacerbation (OR 1.43, 95%CI: 1.16–1.77)	
	Di Raimondo et al. (91)	Case control	40 asthma subjects	40 non-asthma/non-respiratory disease subjects	Asthma ↑ HT prevalence (OR 3.66, CI 1.29–11.1) Severe asthma ↑ HT prevalence (OR 4.32, CI: 1.88–9.54) ↓ %FEV1 ↑ HT prevalence (OR 1.95, CI: 0.96–4.21)	
	Zghebi et al. (92)	Retrospective cohort	3,098,863 weighted asthma admissions		HT ↑ the length of stay for asthma hospitalizations in the observed year of the study	
CVD	Iribarren et al. (93)	Retrospective cohort	203,595 adult asthma subjects	203,595 adult non-asthma subjects	Asthma ↑ risk of all CHD, HR in male and female: 1.28 (CI: 1.21–1.34), 1.49 (CI: 1.43–1.56) respectively Asthma ↑ risk of all cerebrovascular disease HR in male and female: 1.16 (CI: 1.07–1.24), 1.22 (CI: 1.15–1.28) respectively Asthma ↑ risk of heart failure HR in male and female: 2.04 (95%CI: 1.93–2.16), 2.20 (95%CI: 2.10–2.31) respectively Asthma ↑ all-cause mortality HR in male and female: 3.24 (95%CI: 3.04–3.44), 3.29 (95%CI: 3.12–3.47) respectively	The study that investigated the bidirectional impact of CVD (other than HT) on asthma outcomes, including exacerbation, hospitalization, and mortality, and on the success of biologic agents treatment in severe asthma
	Strand et al. (62)	Prospective cohort	14,917 asthma patients	431,429 non-asthma participants	Asthma ↑ CVD mortality HR 1.13 (95%CI: 0.97–1.31), and more prevalent in active asthma HR 1.57 (95%CI: 1.29–1.93) Asthma ↑ CHD mortality HR 1.09 (95%CI: 0.82–1.45), and more prevalent in active asthma HR 1.46 (95%CI: 1.98–2.17) Asthma ↑ stroke mortality HR 1.14 (95%CI: 0.89–1.46), and more prevalent in active asthma HR 1.23 (95%CI: 0.86–1.74)	
	Pelkonen et al. (83)	Cross-sectional	189 hospitalized-asthma + CVD patients	2,399 hospitalized non-asthma + CVD patients	↑ mean hospital days/year because of CVD (asthma vs. non-asthma group: 0.46 vs. 0.20)	
	Ingebrigsten et al. (94)	Prospective cohort	1,168 and 863 early and late-onset asthma patients respectively	40,458 non-respiratory diseases participants	Asthma ↑ risk of hospital admission for CHD both of early onset and late onset group HR 1.24 (95%CI: 0.94–1.64); HR 1.35 (CI: 1.07–1.69) respectively Asthma ↑ risk of hospital admission for heart failure both of early onset and late onset group HR 2.19 (95%CI: 1.43–3.36); HR 1.50 (CI: 1.05–2.15) respectively	

CVD, cardiovascular disease; CKD, chronic kidney disease; CHD, coronary heart disease; DM, diabetes mellitus; ER, emergency room; FEV1, forced expiratory volume in 1 s; FVC, forced vital capacity; GFR, glomerular filtration rate; HT, hypertension; PEFR, peak expiratory flow rate.

### 5.1 Asthma and CKD

CKD represents a substantial global health concern, impacting millions of individuals worldwide. Epidemiological data from 2017 reported a staggering 697.5 million cases of all-stage CKD, with a worldwide prevalence of 9.1%, and 1.2 million CKD-related deaths (95% uncertainty interval: 649.2–752.0) (95). Notably, emerging evidence has linked asthma with CKD, highlighting a bidirectional relationship between asthma and CKD. Individuals diagnosed with asthma have an increased probability of developing CKD, while CKD patients are more prone to asthma development (74, 75, 96, 97). Furthermore, patients with well-controlled asthma displayed a higher glomerular filtration rate than those with uncontrolled asthma, and individuals with severe asthma face an elevated risk of developing CKD than do those with mild/moderate asthma or without asthma, suggesting a potential link between asthma severity and renal function (75, 98).

The impact of asthma on CKD development may be influenced by various other comorbidities prevalent in patients with asthma. High blood pressure, diabetes, obesity, abnormal blood lipid levels, and advancing age are well-known factors that contribute to CKD in the general population (99–102). Additionally, the frequent use of systemic corticosteroids, which is common in asthma treatment, particularly in difficult-to-treat and severe asthma, has been associated with adverse effects, including hypertension and diabetes, which may contribute to CKD risk (103–105). Surprisingly, some studies have suggested a protective effect of corticosteroids against CKD development (74, 106), possibly attributable to their impact on reducing proteinuria and preserving renal function (107, 108).

Asthma is recognized as a chronic airway inflammation, driven by proinflammatory cytokines. However, mounting evidence supports the concept of systemic inflammation extending beyond the lungs in patients with asthma (109). Eosinophilic inflammation, a hallmark of classical asthma, has been linked to CKD development, as peripheral eosinophilia has been associated with a heightened likelihood of end-stage renal failure (110, 111). Another plausible hypothesis involves the link to elevated numbers of neutrophils, which have been implicated in kidney injury. Studies have indicated that individuals with severe asthma tend to have a greater proportion of neutrophils than do those with non-severe asthma (112, 113). Neutrophils have been recognized as independent risk factors for the development and progression of CKD (114, 115). This observation underscores the potential significance of neutrophilic activity in contributing to kidney complications in severe asthma cases. Furthermore, increased levels of C-reactive protein in asthma patients parallel the results observed in individuals with CKD, hinting at systemic inflammation as a potential mechanism contributing to CKD in asthma (116–119).

In summary, asthma, especially in severe cases, appears to contribute to an increased risk of developing CKD. Conversely, CKD also seems to heighten the risk of developing asthma. The current literature encompassing clinical studies on the potential correlation between asthma and CKD remains limited. At present, the potential association between CKD and the severity of asthma, spanning aspects such as poor symptom control, heightened exacerbation rates, increased hospitalization occurrences, response to treatment, and mortality has not yet been conclusively proven. However, understanding the association between these conditions is crucial for effective clinical management and preventive strategies, ultimately contributing to improved patient outcomes. Additional investigation is needed to uncover the underlying connection between asthma and CKD, paving the way for novel therapeutic approaches targeting asthma-related CKD.

### 5.2 Asthma and DM

DM and asthma are two prevalent and chronic health conditions with significant global impact. DM, characterized by elevated blood glucose levels (120), contributes to a range of life-threatening complications, escalating healthcare expenditures, reduced quality of life, and elevated mortality rates (121, 122). Notably, low- and middle-income countries bear the highest burden of type 2 DM (122). Epidemiological estimates reveal a substantial rise in DM prevalence, with projections suggesting an increase from 9.3% in 2019 to 10.9% by 2045, affecting approximately 700 million individuals (123). Concurrently, asthma has demonstrated a significant association with DM, with the prevalence of DM being more elevated among individuals with asthma compared to the general population (124).

Individuals with DM face an increased risk of developing asthma, encompassing both type 1 and type 2 DM, revealing a potential bidirectional relationship between these chronic conditions (76, 77, 79). Furthermore, individuals who have both asthma and DM demonstrate a heightened likelihood of ER visits and hospitalizations, suggesting a role for poor glycemic control in asthma severity (77, 92). Notably, severe asthma has been associated with a higher likelihood of developing type 2 DM, emphasizing the importance of considering asthma severity in assessing DM risk (98). Intriguingly, there appears to be an increased risk of children developing asthma in association with gestational diabetes. However, the precise mechanism underlying this relationship remains unclear and warrants further investigation (125). Evidence from retrospective cohort studies has demonstrated that DM is associated with a rise in overall mortality among individuals with asthma, and higher HbA1c levels in individuals with prediabetes or DM face an increased risk of asthma exacerbations and hospitalizations (78, 80, 81).

Hyperglycemia appears to be intricately linked to impaired lung function, as having a lower forced expiratory volume in 1 s (FEV1) is associated with an increased likelihood of developing DM in the future (126). Moreover, fasting plasma glucose and HbA1c levels exhibit an inverse relationship with lung function test results, indicating the potential impact of glycemic control on respiratory health (127, 128). The development of DM in individuals with asthma could be attributed to systemic corticosteroid administration (98, 106). Numerous studies have investigated the association between corticosteroid use and DM incidence, with higher doses and prolonged administration showing a positive correlation with DM risk (106, 129–131). Nevertheless, certain studies have indicated that there is no notable distinction in the DM incidence between individuals using corticosteroid and non-corticosteroid treatments (132). Additionally, the likelihood of developing corticosteroid-induced DM is affected by various coexisting factors, including age, HbA1c level, and renal function (133). The development of DM is influenced by factors beyond corticosteroid action. This is highlighted by studies demonstrating increased fasting plasma glucose, impaired insulin sensitivity, worsened glucose tolerance, and an incretin effect in healthy individuals following prednisolone administration (134). However, multiple mechanisms underlie the development of corticosteroid-induced DM, encompassing corticosteroid-induced insulin resistance, increased hepatic glucose production, and direct inhibition of insulin release (135, 136).

Interestingly, emerging therapeutic investigations have demonstrated the potential benefits of employing glucagon-like peptide-1 receptor agonists and metformin to reduce asthma exacerbations in individuals who have both type 2 DM and asthma. (137–139). Furthermore, systemic inflammation and pro-inflammatory cytokine expression have been suggested to play a role in the development of both DM and asthma. Individuals with obesity and type 2 DM have significantly higher matrix metalloproteinase-9 and interleukin-4 (IL-4) expression than those without these conditions. IL-4 represents a critical Th2 cytokine responsible for stimulating the production of immunoglobulin E and plays a role in the development of asthma, indicating a possible link between these conditions (140, 141).

This evidence provides critical insights into the intricate relationship between DM and asthma, encompassing prevalence, mechanisms, and potential therapeutic interventions. Conclusively, DM is associated with adverse asthma outcomes, encompassing increased asthma development, exacerbation rates, higher rates of hospitalization, and impaired lung function. It has been observed that strategic interventions involving anti-diabetic pharmacological treatments can effectively reduce the risk of exacerbations in individuals who have both asthma and diabetes. Moreover, it highlights the significance of clinician awareness regarding the use of corticosteroids in asthma management, taking into consideration the associated risk of developing DM. The exploration of common pathophysiological pathways between these conditions opens avenues for targeted and personalized treatment approaches, aiming to alleviate the burden of DM and asthma on affected individuals and healthcare systems globally. Future research should focus on deciphering the underlying mechanisms linking DM and asthma. Future research should prioritize the discovery of the fundamental mechanisms connecting DM and asthma, as well as assessing the impact of DM on the response to biologic agents in the management of severe asthma patients, to develop effective preventive strategies and optimize patient outcomes, particularly the impact of diabetes on the success of biological agent administration in severe patients with asthma.

### 5.3 Asthma and lung cancer

Lung cancer remains a major global health issue, responsible for a significant proportion of cancer cases and deaths worldwide. According to the Global Cancer Statistics 2020, lung cancer is the second most prevalent cancer, accounting for 11.4% of all cancer diagnoses, and it continues to be the primary contributor to cancer-related fatalities, causing an estimated 1.8 million deaths attributed to the disease. Remarkably, lung cancer is more prevalent among men, representing the most prevalent form of malignancy and the leading cause of cancer-related mortality in the male population (142).

Evidence indicates a favorable correlation between asthma and lung cancer, as numerous studies have documented a heightened incidence and an elevated likelihood of lung cancer has been observed in individuals with asthma (82, 84, 143–148). Notably, active or partially controlled asthma appears to elevate the probability of developing lung cancer (149). Importantly, the association between asthma and lung cancer is more pronounced in smokers (150), suggesting an interaction between smoking and asthma in lung cancer development. Nevertheless, asthma that necessitates medical management is also autonomously associated with a notably increased prevalence of lung cancer in non-smoker individuals, highlighting the multifaceted nature of this relationship (147).

Within the context of hospitalization for lung cancer, asthma has emerged as a significant determinant, contributing to an increased mean number of hospital days per year, as compared to individuals without asthma (83). This observation underscores the potential influence of asthma on the overall healthcare burden associated with lung cancer. Additionally, a history of asthma is related to particular histological subtypes of lung cancer, with squamous cell carcinoma being more common in this population (145). However, in clinical practice, it cannot be solely relied upon to suggest the histological subtype diagnosis of lung cancer because some studies have shown an increased risk of all major lung cancer subtypes, including squamous cell carcinoma and adenocarcinoma, in both men and women with asthma (151).

The asthma control test (ACT) score, reflecting the effectiveness of asthma control, may be indicative of lung cancer progression. At the time of lung cancer diagnosis, individuals with asthma who were diagnosed with lung cancer experienced a deterioration in their symptoms as indicated by their ACT scores at the time of lung cancer diagnosis compared to the scores from the previous year. Furthermore, an improvement in ACT scores can be observed in lung cancer patients in the complete, partial response, and stable disease groups after effective lung cancer treatment, while those with progressive disease show worsened scores (152).

The majority of pharmacological treatments for asthma, including ICS, inhaled antimuscarinics, theophylline, antihistamines, and short and long-acting beta-agonists, do not appear to be associated with an increased risk of cancer in patients with asthma. Nonetheless, the use of oral corticosteroids is associated with a slight elevation in risk (146). Notably, an animal study has shown that dexamethasone may inhibit tumor cell proliferation and prevent cell cycle progression by lowering proliferating cell nuclear antigen and cyclin D1 expression, suggesting the possibility of corticosteroids playing different roles in the development of lung cancer (153).

Being diagnosed with lung cancer has been related to a higher risk of mortality in male asthma patients (58). However, the prognosis evaluated by overall survival rates for lung cancer patients, whether they have asthma or not, do not show significant differences. A study observed that the 5-year cumulative corrected survival rates for lung cancer are similar between asthma-lung cancer patients and non-asthma-lung cancer referents, at 8.4% and 9.6%, respectively (154).

Regular use of ICS may offer protection against lung cancer. A cohort study found that individuals with mild asthma who regularly used ICS had a lower lung cancer risk than those with severe asthma who did not use ICS regularly (155). This beneficial impact is also evident in individuals with COPD, wherein ICS exposure is linked to a decreased likelihood of being diagnosed with lung cancer (156). The precise mechanism underlying this protection is currently unknown but may involve ICS-mediated improvements in airway remodeling and inflammation through the regulation of matrix metalloproteinases, which are key proteins associated with tumorigenesis (157–160).

Asthma should be regarded as a possible adverse reaction when administering immune checkpoint inhibitors (ICIs) to treat lung cancer. A case report documented the development of asthma symptoms in a lung cancer patient following durvalumab administration after chemoradiotherapy (161). Durvalumab-induced pneumonitis and radiation pneumonitis could potentially contribute to these symptoms (162). The precise mechanism remains unclear, but it may be related to an elevation in pro-inflammatory cytokines while undergoing concurrent chemo-radiation therapy. Radiotherapy has been demonstrated to elevate the levels of IL-6 in the bronchoalveolar lavage fluid of individuals with lung cancer (163), while changes in expression of cytokines, such as fractalkine/CX3CL1, granulocyte-monocyte colony-stimulating factor, IL-1α, interferon-γ (IFN-γ), interferon-inducible protein-10, macrophage inflammatory protein-1β, soluble CD40 ligand, and vascular endothelial growth factor (VEGF) have also been observed during various types of radiotherapy (164), providing further evidence of inflammatory processes following radiotherapy administration.

Taken together, previous research has provided evidence supporting the role of asthma in contributing to an increased incidence, hospitalization rates, and mortality associated with lung cancer. Nevertheless, additional investigations aimed at assessing the noticeable effect of lung cancer on outcomes associated with asthma, including the severity of symptom control, frequency of exacerbations, response to treatment, and mortality, are necessary. The complex relationship between asthma and lung cancer requires careful consideration in clinical practice. Understanding the interplay between these two conditions may aid in early detection, risk stratification, and tailored therapeutic approaches for patients affected by both asthma and lung cancer.

### 5.4 Asthma and obesity

Obesity persists as a significant global health issue, with age-standardized estimates of DALYs related to obesity reaching 1933 (95% UI: 1,277–2,640). The annual increase in obesity-related DALYs was 0.48% from 2000 to 2019, and it is projected to surge by 39.8% from 2020 to 2030 (165). Obesity and overweight also significantly affect societal expenses including annual medical care costs and the quality of life (166, 167). Additionally, excessive weight was also accountable for a rise in mortality, causing over 1,300 additional deaths daily (almost 500,000 annually) and contributing to a decrease in life expectancy (168). This underscores the escalating impact of obesity on the burden of disease, emphasizing the urgent need for comprehensive strategies to address and mitigate its consequences.

Obesity is associated with an increased incidence of asthma (85, 169, 170). Interestingly, individuals with asthma are more prone to developing obesity when compared to those without asthma. More specifically, adults with asthma, particularly those who are non-atopic, have a longer duration of the disease or are using oral corticosteroids (171). The bidirectional relationship is evident not only in adults but also in childhood and adolescence, indicating that the development of asthma is influenced by childhood obesity (172, 173), creating a complex interplay between these health conditions. Individuals who simultaneously suffer from asthma and obesity are more prone to experiencing poorly controlled asthma symptoms (86, 174–176) and lower quality of life (176). Furthermore, individuals in this context demonstrate reduced responsiveness to asthma medications, as evidenced by a higher likelihood of utilizing asthma medications such as short-acting β2-agonists, frequently dispensed and OCS use (177), increased doses of ICS (178, 179), a heightened risk of exacerbations (14, 180), increased emergency room visits and a higher likelihood of hospitalization, including extended hospital length of stay and a higher risk of mechanical ventilation (68, 87, 177, 181). The impact of obesity extends further, contributing to elevated mortality rates among individuals with asthma (88).

Obesity exerts a notable influence on lung function. Obese individuals exhibited a reduction in several measures of lung function, demonstrating a pattern of lower values for FEV1, FVC, TLC, and RV (182, 183). In the general non-asthmatic population, body mass index (BMI) demonstrates a negative correlation with forced expiratory volume in 1 s (FEV1) and forced expiratory flow between 25% and 75% of vital capacity (FEF25-75%) in obese subjects (184). These findings align with those observed in the asthmatic population, where individuals with asthma who are obese demonstrated lower FEV1 and FVC compared to patients without obesity (183, 185). In a prospective study, it was noted that an increase in BMI is linked to a more rapid decline in FEV1 and FVC among adults with asthma who are overweight or obese when compared to those of normal weight (186).

Obesity is associated with poorer clinical outcomes in asthma patients, although the exact mechanisms underlying this connection remain limited in understanding. One potential explanation is that obesity serves as a risk factor for various chronic diseases that can manifest alongside asthma, exacerbating the overall health condition. Numerous studies provide evidence indicating that obesity is linked to a significant increase in various inflammatory markers, potentially intensifying the inflammatory processes in individuals with asthma. Asthmatic individuals who are obese demonstrate notably elevated levels of inflammatory markers, including blood and sputum neutrophils (176, 187), bronchial sputum eosinophil count (187), sputum IL-5 (187), blood IL-6 (187, 188), CRP (187, 189, 190), TNF-α (188), and leptin (187, 188, 190–192), in comparison to their non-obese counterparts with asthma. Additionally, obese asthma patients often exhibit a distinct phenotype characterized by neutrophilic inflammation. This is evident in the observed correlation between neutrophil levels and the BMI parameter (193, 194).

The concept of the “obesity paradox,” which suggests that clinical outcomes are more favorable in overweight or obese patients than in those with normal weight or underweight, is widely recognized across various diseases like heart failure (195–198), chronic kidney disease (199–201), peripheral arterial disease (202), myocardial infarction (203–206), lung cancer (207–209), and COPD (210–212). This paradoxical phenomenon is evident in the observation that, despite the common belief that excess weight may be detrimental to health, it seems to confer certain advantages in terms of clinical outcomes in specific medical conditions. The concept of the obesity paradox in asthma remains unclear and not fully understood. A study discovered that individuals categorized as pre-obese experience a lower risk of mortality due to all causes and respiratory diseases when compared to those in the normal-weight group. Nevertheless, the long-term impact of obesity on outcomes in asthma remains uncertain due to the limited number of obese patients included in this study (213).

In summary, the influence of obesity on clinical outcomes in asthma has been extensively documented in various studies, affecting factors such as poor asthma control, exacerbations, diminished lung function, increased hospitalization rates, and mortality. To enhance clarity, there is a requirement for a prospective study and clinical investigations specifically focusing on the impact of obesity on airway remodeling in asthma. Despite the existence of the obesity paradox in certain respiratory diseases like lung cancer and COPD, this phenomenon in the context of asthma remains relatively less understood to date.

### 5.5 Asthma and CVD

CVD stands as the predominant cause of morbidity and mortality on a global scale, presenting a significant challenge to public health worldwide. Over the past three decades, there has been a noteworthy surge in the overall prevalence of CVD, escalating from 271 million to 523 million by the year 2019. Simultaneously, the number of deaths attributed to CVD has demonstrated a persistent upward trajectory, rising from 12.1 million in 1990 to 18.6 million in 2019 (214). In contrast to other forms of cardiovascular disease (CVD), ischemic heart disease and stroke constituted 85% of the entire age-standardized death rate (ASDR) for CVD. Both the ASDR and age-standardized DALYs rate (ASYR) for CVD were 1.5 times higher in men than in women. Individuals aged 50 and above were particularly vulnerable to CVD development, with this age category contributing to over 90% of all cases and deaths across various age groups (215). Hypertension, a modifiable risk factor for other significant cardiovascular diseases like heart failure (216, 217), stroke (218, 219), and ischemic heart disease (220), demands attention. The approximate worldwide prevalence of hypertension in 2000 was recorded at 972 million adults, and projections anticipate an increase to 1.56 billion by the year 2025 (221). These alarming numbers highlight the urgent need for effective interventions and preventive measures to mitigate the impact of CVD on global mortality.

Asthma has been identified as a prevalent chronic comorbidity and risk factor for CVD, including hypertension, heart failure, ischemic or coronary heart disease, stroke, pulmonary embolism, deep vein thrombosis, and peripheral artery disease (222–228). This association may be more pronounced in the adult-onset asthma (229). Asthma not only coexists with CVD but may also contribute, increasing the likelihood of significant cardiovascular events. Numerous studies observed that asthma is linked to a heightened risk of coronary heart disease (93, 230), heart failure (93), and stroke (93, 227, 231–233), increased risk of hospital admission and mean hospital day because of CVD (83, 94), as well as an elevated risk of all-cause mortality and CVD morbidity and mortality, especially in current asthma (62–64, 93). This increased risk is not limited to chronic conditions. A study carried out in an Asian population demonstrated that asthma was correlated with a higher probability of encountering acute coronary syndrome, particularly in older individuals with extended emergency room visits or the need for hospitalization (234).

In the context of a bidirectional relationship between CVD and asthma, hypertension emerges as a potential risk factor for asthma. Evidence indicates that individuals with high blood pressure are more susceptible to developing asthma, with severe asthma cases exhibiting higher odds ratios (79, 91). Conversely, individuals with asthma tend to exhibit elevated blood pressure levels when compared to those without asthma and the prevalence of elevated blood pressure is notably higher in individuals with asthma who have lower FEV1% (89). Moreover, hypertension, along with ischemic heart disease and cerebrovascular events, increases the risk of uncontrolled asthma (235, 236). Hypertension also has an impact on a decline in lung function (237), heightened severity of asthma exacerbations and prolonged hospital stays (90, 92), posing challenges in asthma management and necessitating tailored therapeutic approaches. This observation highlights a significant potential association between asthma and hypertension, suggesting that the presence of asthma may contribute to alterations in blood pressure. While hypertension has been well-defined as a risk factor for asthma, other forms of cardiovascular diseases, such as heart failure, ischemic heart disease, and stroke, do not appear to serve as risk factors for the development of asthma and have a limited impact on asthma outcomes. This distinction underscores the complexity of the relationship between cardiovascular health and asthma, indicating that the impact of specific cardiovascular conditions on the risk of asthma may vary. The relationship between asthma and CVD may be attributed to the inherent nature of each condition. The mechanism by which hypertension induces asthma may involve angiotensin II-induced bronchial smooth muscle hyperresponsiveness through the activation of p42/44 ERK (238). However, it remains unclear whether heart failure or other cardiovascular diseases accompanied by hypertension induce asthma, both in laboratory and clinical research. Further investigation is needed to elucidate the specific pathways and interactions contributing to the development of asthma in the context of various cardiovascular conditions associated with hypertension.

Clinical risk stratification is a crucial strategy for identifying individuals at high risk of future cardiovascular events and implementing optimal preventive measures. By assessing various risk factors and considering an individual’s medical history, lifestyle, and other relevant factors, clinicians can tailor preventive strategies to the specific needs of each patient. The European Society of Cardiology Systematic Coronary Risk Evaluation (SCORE), the American College of Cardiology/American Heart Association Atherosclerotic Cardiovascular Disease (ASCVD) risk assessment tools, and the Framingham risk score can be employed to evaluate the risk of cardiovascular disease (239, 240). Conversely, there is a growing interest in utilizing pulmonary function testing to predict cardiovascular disease risk stratification in the context of asthma. A decline in lung function, indicated by reductions in FEV1/FVC, FVC, and FEV1, has been observed to be associated with an elevated risk of cardiovascular (241–245). Long-term cohort studies conducted among older individuals have indicated that there are inverse relationships between FEV1 and FVC and the likelihood of experiencing future events related to CVD, highlighting the potential utility of pulmonary function tests in predicting cardiovascular outcomes in older individuals (246). The utilization of pulmonary function tests to predict CVD in clinical practice is not yet widely adopted. However, clinicians should be attentive to the potential for cardiovascular events in asthmatic patients who exhibit a decline in lung function. The assessment of combined CVD risk stratification and pulmonary function tests may play a role in predicting cardiovascular events in asthma patients, although further research is needed to fully understand and validate this relationship.

Another aspect that requires consideration is drug administration in both asthma and CVD. The use of β-blockers in individuals with both hypertension and asthma demands careful attention due to the risk of bronchoconstriction. Non-selective β-blockers like propranolol, pindolol, and timolol have the potential to induce bronchoconstriction in asthma patients (247–249). Conversely, cardio-selective β-blockers were observed to pose a reduced risk of exacerbations (250), suggesting a more favorable therapeutic profile.

Another crucial consideration involves evaluating the cardiovascular safety profile of biologic agents used in severe asthma patients. The EXCELS cohort study has provided valuable insights, revealing that omalizumab is associated with a higher incidence of cardiovascular and cerebrovascular serious adverse events when compared to non-omalizumab-treated patients. The observed rates of such events were 13.4 per 1,000 person-years for omalizumab-treated individuals, as opposed to 8.1 per 1,000 person-years in the non-omalizumab group. Furthermore, the study highlighted an increased risk of arterial thromboembolic events among those receiving omalizumab, with rates of 6.66 per 1,000 person-years, compared to 4.64 per 1,000 person-years in the non-omalizumab cohort (251). COSMOS, an extension derived from the MENSA and SIRIUS studies observed no fatal serious adverse events were documented throughout this study. However, in the previous mepolizumab group, 9 patients (2%) reported experiencing any cardiovascular events (252). Benralizumab appears to demonstrate a favorable safety profile in cardiovascular aspects, as evidenced by findings from the SIROCCO and BORA (253, 254). Similarly, a separate study on dupilumab yielded comparable results. Across these trials, there were no reported adverse events linked to cardiovascular issues (255, 256). Upon analyzing data from the PATHWAY and NAVIGATOR studies, notable findings emerged concerning cardiovascular events. Serious adverse events related to cardiac disorders were reported at rates of 0.8% and 0.3% in the tezepelumab and placebo groups, respectively. Additionally, the incidence of hypertension was observed at 3.9% and 4% in the tezepelumab and placebo groups, respectively (257). Continued research dedicated to evaluating the cardiovascular safety profile is imperative. Clinicians must exercise caution and consideration, particularly when managing severe asthma patients with cardiovascular comorbidities using biologic agents. This proactive approach ensures a more comprehensive understanding of the potential implications and aids in making informed decisions for personalized and effective asthma treatment.

In summary, asthma is recognized as a well-defined risk factor for CVD, although the bidirectional relationship between the two is not fully understood. Specifically, among CVD, hypertension is the only condition with a clear impact on asthma outcomes, affecting asthma control, exacerbation rates, lung function decline, and hospitalization. Further studies are required to explore the potential impact of other CVDs on asthma outcomes. The use of pulmonary function tests in combination with CVD risk stratification may play a role in predicting CVD in asthma patients. When considering medications for individuals with both asthma and CVD, a careful evaluation of risks and benefits is essential.

### 5.6 Asthma as part of ACO

**Figure 2:**
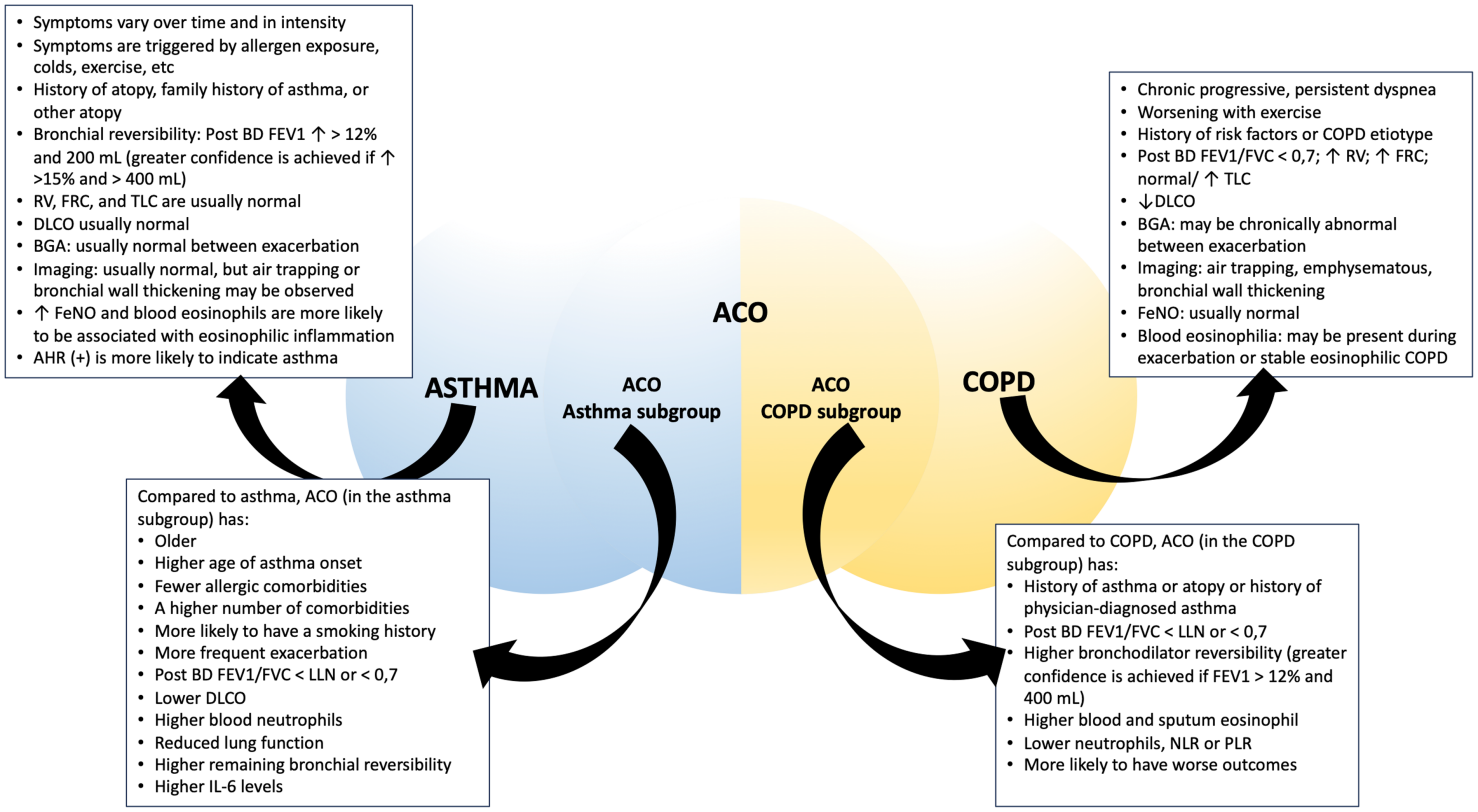
Comparison between asthma, COPD, and ACO. Abbreviation: post-BD: post-bronchodilator; FEV1, forced expiratory volume in 1 s; RV, residual volume; FRC, functional residual capacity; TLC, total lung capacity; DLCO, diffusing capacity for carbon monoxide; BGA, blood gas analysis; FeNO, fractional exhaled nitric oxide; AHR, airway hyperresponsiveness; FVC, forced vital capacity; LLN, lower limit normal; NLR, neutrophil-to-lymphocyte ratio; PLR, platelet-to-lymphocyte ratio.

Asthma is a complex and multifaceted respiratory condition that can manifest in various respiratory problems and is often accompanied by a range of comorbidities. Among these comorbidities, particularly notable in older individuals previously diagnosed with asthma, is Asthma-COPD Overlap (ACO). ACO is not defined as a single entity; rather, it is a condition characterized by the coexistence of features typically associated with both asthma and COPD. Establishing precise diagnostic criteria for asthma, ACO, and COPD within clinical practice is a complex task. The presence of overlapping clinical manifestations and shared characteristics among these respiratory conditions can introduce significant challenges to the diagnostic process. These challenges may include differentiating between the conditions based on symptoms, lung function tests, and clinical histories (Figure 2). To provide effective care and individually tailored treatments, clinicians must navigate this complexity by carefully evaluating the unique features of each condition, while also recognizing the potential for overlap. Accurate diagnosis is crucial to ensure that patients receive the most appropriate interventions and therapies, improving their overall respiratory health and quality of life. Asthma, COPD, and ACO all fall under the classification of obstructive pulmonary disease. According to GINA, ACO is used as a collective description of individuals who exhibit persistent airflow limitation along with clinical characteristics that correspond to both asthma and COPD (1).

The prevalence of ACO can differ significantly depending on the diagnostic criteria employed in the clinical study. A prospective study observed ACO prevalence ranging from 3.8% to 31% (258). Additionally, another study that utilized self-reported data and analyzed different age groups reported ACO prevalence rates of 1.9% in the general population. In different sub-groups, the prevalence was 0.7% in the <40-year age group, 1.4% in the 40- to 60-year age group, and 3.2% in the ≥60-year age group (259). This study also revealed that the prevalence of ACO was 2% after evaluating ACO using spirometry parameters and including the criteria of doctor-diagnosed asthma (259). A meta-analysis revealed that the prevalence of ACO in the general population is relatively low at 2%. However, in populations of individuals with asthma and COPD, the prevalence is significantly higher, at 26.5% and 29.6%, respectively. These findings emphasize the importance of clinical awareness when managing ACO, asthma, and COPD in clinical practice, especially in older patients with a history of asthma (260).

Asthma exhibits variable patterns of disease progression, with symptoms ranging from intermittent to persistent. While some individuals achieve long-term asthma control through diligent management and adherence to prescribed therapies, certain risk factors may contribute to the potential transition to ACO over time. ACO is caused by a dynamic interaction between a host and environmental factors of which the influence begins during fetal development and continues into adulthood. Factors such as continued exposure to occupational or environmental triggers, sustained smoking habits, genetic susceptibility, aggravation of symptoms, interaction among inflammatory cells, and the presence of comorbidities, among others, have been suggested as contributors to the development of ACO in individuals with a history of asthma (261–264). Epigenetic factors associated with smoking/allergen exposure may play an important role in the development of ACO. A study employing microarray analysis has made notable observations regarding the epigenetic factors contributing to the development of ACO. It identified specific genes that undergo hypermethylation or hypomethylation in individuals with ACO, distinguishing them from those with COPD or healthy non-smokers. Moreover, this study found that these epigenetic changes were associated with rapid declines in lung function, severe airflow limitations, and frequent exacerbations (265).

Establishing accurate diagnostic criteria for both asthma and ACO within clinical practice presents a challenging endeavor. The overlapping clinical manifestations and shared characteristics between these conditions can complicate the diagnostic process. The hallmark of asthma is characterized by variable expiratory airflow limitation assessed through lung function tests or airway hyperresponsiveness. ACO, on the other hand, exhibits clinical presentations that can resemble asthma, primarily because it combines features of both asthma and COPD, encompassing a wide range of clinical presentations. Identification of specific diagnostic markers to distinguish between these entities remains an ongoing research focus. Sputum cell counts hold potential as inflammatory biomarkers. Sputum eosinophil (%) is reduced in COPD as compared to asthma and ACO, while sputum neutrophil (%) is elevated in COPD. Conversely, sputum macrophage (%) is elevated in patients with asthma (266). Another common marker that can be used to differentiate between these three obstructive lung diseases is blood eosinophil levels, along with the analysis of complete blood count. ACO showed elevated eosinophil counts, reduced percentages of neutrophils, a decreased neutrophil-to-lymphocyte ratio, and a lower platelet-to-lymphocyte ratio (267). In a population-based, cross-sectional study, it was observed that individuals with ACO had a higher prevalence of blood eosinophil counts exceeding 400 cells/μL when compared to those with COPD or asthma (268). Nevertheless, it’s essential to recognize that elevated blood eosinophil levels are not unique to ACO, as another study demonstrated that asthma patients tend to have higher blood eosinophil counts than both ACO and COPD patients (269). This variation in findings may be because individuals in the ACO group can exhibit characteristics that align more closely with either COPD or asthma (268). Serum nuclear magnetic resonance spectroscopy demonstrated promise in differentiating asthma, COPD, and ACO. It revealed dysregulated metabolites such as lipids, isoleucine, N-acetyl glycoproteins, glutamate, valine, glucose, citric acid, l-leucine, lysine, phenylalanine, asparagine, and histidine, which were found to be distinctive markers for ACO, setting it apart from asthma and COPD (270).

Micro-RNA, particularly miR-15b-5p, stands out as a potential candidate for precise ACO identification. Moreover, when miR-15b-5p is combined with YKL-40 and periostin, it has proven to be useful in defining the characteristics of ACO, providing insights into its pathophysiological nuances (271). Additionally, the assessment of damage-associated molecular patterns has highlighted significant differences across these conditions. Sputum high mobility group protein B1 levels were elevated in individuals with ACO and with COPD, as compared to those with asthma, while sputum LL-37 levels were higher in individuals with COPD than in those with ACO (272). Fractional exhaled nitric oxide (FeNO) serves as a straightforward, safe, and quantitative means of evaluating airway inflammation. It quantifies the levels of nitric oxide (NO) produced by nitric oxide synthase during exhalation (273). FeNO levels can be utilized to distinguish between ACO and COPD (274, 275). However, the utility of FeNO in distinguishing between asthma and ACO remains unclear at present. This is because ACO often presents with characteristics of eosinophilic airway inflammation that are similar to those seen in asthma. Since exhaled nitric oxide levels have been found to have a positive correlation with eosinophilic sputum (276), higher FeNO levels may indicate increased eosinophilic inflammation in the airways. However, it’s important to note that elevated FeNO levels are not specific to ACO or asthma alone.

An essential aspect of effectively managing asthma, COPD, and ACO is the ability to distinguish between these conditions. When comparing the ACO-asthma subgroup to the ACO-COPD subtype, several notable differences emerge. The ACO-COPD subgroup tends to consist of older individuals, with a higher proportion of males and a greater prevalence of smoking history. Additionally, these individuals often exhibit compromised lung function and an increased susceptibility to exacerbations (277–279). Investigation within a population with asthma and severe asthma has emphasized that ACO patients are generally advanced age, predominantly male, and more prone to having a history of smoking, a higher mean age of asthma onset, longer disease duration, less prevalence of allergic comorbidities, poorer lung function measurements, and a requirement for higher doses of ICS (280–282). Furthermore, ACO patients have lower diffusing capacity of carbon dioxide (DLCO), higher blood neutrophils, are more likely to have higher sputum neutrophil levels, higher IL-6 levels, increased bronchial reversibility, and a greater number of comorbid conditions (282, 283). On the other hand, within a population with COPD, ACO patients exhibit higher bronchodilator reversibility, a medical history of either asthma or atopic conditions, and higher blood and sputum eosinophil levels (268, 282). In another study conducted within the COPD population, individuals with ACO exhibited a more unfavorable prognosis. They displayed lower lung function and a higher likelihood of being fast decliners. ACO patients experienced more severe symptoms and a greater frequency of exacerbations compared to other COPD patients. They also had a higher burden of comorbidities and reported impaired quality of life. Additionally, features such as atopy and physician-diagnosed asthma were found to be more distinctive for identifying ACO (258).

Individuals with COPD are more likely to experience hospitalization due to the progressive nature of the disease, worsening clinical symptoms, and a decline in lung function, particularly in those with frequent exacerbations. Similarly, individuals with ACO also demonstrate a heightened hospital impact compared to patients with asthma. Both COPD and ACO patients experience more adverse outcomes and a greater need for hospital care when compared to asthma patients (284). The higher rate of hospitalization in ACO patients may be attributed to several factors, including the progressive nature of the disease, worsening clinical symptoms, a decline in lung function, higher rates of fast decliners, increased symptom burden, more frequent use of systemic corticosteroids, greater occurrence of exacerbations and ER visits, compromised health-related quality of life, and a higher prevalence of comorbidities (258, 280, 285, 286).

Asthma, COPD, and ACO all contribute to an increased risk of mortality. However, understanding the comparative mortality rates among these three conditions remains complex. While each of these respiratory conditions has its unique characteristics and risk factors, overlapping symptoms and comorbidities often blur the lines between them. As a result, determining the precise mortality risk associated with each condition, as well as identifying the specific factors that influence mortality, poses significant challenges. Utilizing a national inpatient database, a comprehensive study has assessed the in-hospital mortality for all causes in patients admitted for exacerbations of asthma, COPD, or ACO. The findings of that study revealed distinct patterns: the group exclusively affected by COPD exhibited the highest in-hospital mortality rate, whereas asthma demonstrated comparatively lower in-hospital mortality than the ACO group (287). Furthermore, individuals with ACO present an elevated risk of death related to respiratory issues, in contrast to those experiencing asthma alone, without a corresponding trend observed among those exclusively affected by COPD. In investigating cause-specific mortality, COPD and asthma predominantly contribute to cardiovascular- and cancer-related deaths, respectively, while ACO is characterized by a higher rate of respiratory deaths (288).

Taking together, it is crucial to evaluate other obstructive conditions, such as ACO or COPD, in older individuals with a history of previous asthma. Identifying ACO within populations of asthma or COPD patients is essential for improving the management of these conditions. It has been firmly established that ACO is associated with worse clinical outcomes, including clinical presentation, exacerbations, declining lung function, and mortality when compared to asthma alone. While the management approach for the ACO-asthma subgroup is not significantly different from that of asthma, it necessitates increased awareness, as individuals with ACO may present with multiple comorbidities that can impact the success of treatment. Future research should prioritize the development of biomarkers to distinguish between asthma and ACO and the elucidation of personalized management strategies for individuals clinically diagnosed with ACO, especially those with a history of previous asthma.

### 5.7 Asthma as part of occupational lung disease

Occupational exposure should be recognized as a potential risk factor for respiratory diseases, including asthma. According to a surveillance report from SWORD (Surveillance of Work-related and Occupational Respiratory Disease), there is a discernible upward trend in the incidence of occupational lung disease, with numerous substances impacting respiratory health. SWORD ‘99 highlights that benign pleural disease, occupational asthma, and mesothelioma maintain high incidence rates, constituting 28%, 26%, and 23%, respectively. SWORD ‘98 previously identified occupational asthma as the most reported occupational disease (289, 290).

Based on the underlying mechanisms that trigger asthma symptoms, work-related asthma can be categorized into two main types: occupational asthma, primarily caused by exposure in the workplace, and work-exacerbated asthma, which denotes the onset or exacerbation of asthma symptoms due to occupational exposure in individuals with pre-existing asthma conditions. Occupational asthma can further be classified into sensitizer-induced and irritant-induced occupational asthma (291, 292).

Focusing on occupational asthma, an analysis of several surveillance systems in Korea reveals that the annual mean incidence rate of occupational asthma is 1.6% and 3.5% by compensation and by the surveillance system, respectively (293). Another Korean surveillance study observed the highest incidence of work-related asthma in the 50–59 age group, with the most common agents being isocyanates, flour/grain, metal, reactive dyes, and solvents, accounting for 46.6%, 8.5%, 5.9%, 5.1%, and 4.2%, respectively (294). The incidence of occupational asthma in Korea appears likely lower than in other countries. For instance, a retrospective review of Belgian workers observed an annual incidence of occupational asthma at 29.4 new cases per million workers during the study period, with the most common agents being flour/grain, isocyanates, latex, wood dust, and enzymes, accounting for 33.6%, 19.6%, 17.2%, 7.8%, and 4.8%, respectively (295). Another study, an analysis of data from a 15-year study by Shield Surveillance in the United Kingdom, observed that the annual incidence of occupational asthma was 42 per million workers. This study also noted that isocyanates were the most common agents responsible for occupational asthma (296).

Occupational exposure appears to be correlated with the severity of asthma, and awareness should be heightened regarding moderate to severe adult-onset asthma. An epidemiological study noted an association between severe adult-onset asthma and occupational asthma triggers, encompassing high molecular weight agents, low molecular weight agents, and mixed environments (297). In the context of hospitalization among workers, occupational asthma seems to be linked to a higher likelihood of hospitalization for various reasons, such as cardiovascular disease, respiratory disease, and asthma itself (298). However, it is important to note that this study was conducted in an earlier era when anti-inflammatory agents were not widely utilized for achieving well-controlled asthma. However, in a population-based asthma cohort study, a relatively high incidence of exacerbation, approximately 26%, was observed among asthmatic workers. More than two-thirds of these individuals experienced moderate to severe exacerbations. The study also identified several agents associated with exacerbation in asthmatic workers, including exposure to any gas, smoke, or dust, including organic dust, as well as factors such as cold, dampness, molds, and engaging in physically strenuous jobs (299). A retrospective cohort study identified several factors associated with the severity of conditions in occupational asthma. These factors include prolonged exposure to causative agents at the workplace, childhood-onset asthma, limited educational attainment, and the presence of sputum production (300).

Identifying asthma risk factors and workplace agent exposures that contribute to symptoms and exacerbations is crucial and well-established in the management of occupational asthma. However, clinicians sometimes overlook the identification of comorbidities that accompany occupational asthma and may exert an influence on the uncontrolled condition. While studies on comorbidities in occupational asthma are limited, particularly in terms of how comorbidities influence the outcome of occupational asthma, several studies and case reports have reported the presence of comorbidities in occupational asthma. These include psychiatric disorders such as mood and anxiety disorders and hypochondriasis (301), as well as occupational rhinitis (302, 303). Interestingly, one report observed the development of silicosis in several artificial stone workers who had a prior diagnosis or suspicion of work-related asthma, raising awareness about the potential for more serious conditions to arise from chronic exposure to causal agents (304). Clinicians should be aware of comorbidities in occupational asthma as an integral component of asthma management to achieve well-controlled outcomes. In a case report detailing uncontrolled occupational asthma, a persistent condition was observed despite avoiding the triggering agent and undergoing optimal treatment. However, addressing two comorbidities—GERD and OSA—in this case report led to an improvement in the patient’s condition (305). This underscores the importance of a comprehensive approach that considers and manages associated comorbidities for the effective control of occupational asthma.

In summary, addressing occupational exposure is crucial as a causal factor in the development of occupational asthma among workers. Exposure to causal agents in the work environment appears to be linked with the severity of asthma, including the likelihood of exacerbations. Regarding comorbidities in occupational asthma, while the research in this area is limited, clinicians should be mindful of this issue. Optimal management of comorbidities holds the potential to enhance overall control and achieve a well-managed condition in occupational asthma. Recognizing and addressing both occupational exposure and associated comorbidities constitute essential components of a comprehensive approach to managing occupational asthma effectively.

## 6 Future perspective

Personalized therapy is a future goal in asthma management. The assessment of comorbidities in each individual and the implementation of optimal approaches and management are crucial components of personalized asthma management. The impact of comorbidities extends beyond the outcomes of asthma treated with standard bronchodilators and/or inhaled corticosteroids (ICS) alone. It is also associated with the outcomes achieved through the use of biologic agents in cases of severe asthma. The study observed that severe asthmatic patients with comorbid chronic rhinosinusitis, with or without nasal polyps, showed a significant reduction in exacerbations per year, higher chances of improved post-biologic treatment, and a noteworthy increase in FEV1 compared to those without this comorbidity. Similarly, patients with nasal polyps experienced a decrease in exacerbations and higher odds of improved post-biologic treatment, but there was no significant trend towards additional improvement in FEV1. Conversely, biologic treatments for either chronic rhinosinusitis with or without nasal polyps or nasal polyps did not lead to further reductions in the daily dose of long-term oral corticosteroids. When assessing the impact of allergic rhinitis or atopic dermatitis on the response to biologic agents, no distinct effects on treatment outcomes were observed (306, 307). Conducting studies to investigate the influence of comorbidities on severe asthma patients receiving biologic agents is crucial for future research. This will contribute to an enhanced understanding of asthma-related comorbidities and their impact on treatment outcomes.

While CKD appears to be linked to asthma development, its impact on asthma outcomes requires further investigation. Additionally, the long-term effects of asthma treatments, including biological agents, on renal function warrant attention. DM is well-established as being associated with asthma outcomes. Although studies indicate that the appropriate use of ICS in asthma does not trigger DM, the prolonged use of high-dose ICS or OCS poses the risk of new-onset DM or an increase in blood glucose levels or HbA1C. Therefore, further research is needed to provide appropriate guidance on the use of ICS or OCS in asthmatic patients with DM. Asthma is recognized as an adverse outcome factor in lung cancer, but the influence of lung cancer on asthma outcomes necessitates further investigation. The existing treatments for asthma, including the use of biological agents, have not definitively been shown to increase the incidence of lung cancer. However, prospective studies, particularly examining the use of biological agents in asthma cases and their impact on the incidence of lung cancer, are needed. Moreover, investigations into the use of immune checkpoint inhibitors in lung cancer cases with asthma are also warranted. The bidirectional relationship between asthma and obesity is well-established in numerous studies, and management strategies for asthma in obesity have been extensively discussed in the literature. However, the implementation of these strategies must be individualized for each patient, requiring careful attention from clinicians. Asthma is well-established as a risk factor for various CVD. Nonetheless, the influence of CVD on asthma outcomes remains a subject that requires further study. Additionally, there is a need for studies on cardiovascular risk stratification in asthma patients with reduced lung function.

## 7 Conclusion

In conclusion, the substantial impact of chronic comorbidities necessitates further investigation. CKD heightens the risk of asthma, yet its influence on asthma outcomes remains unclear. Clinicians should remain vigilant regarding CKD development, especially in severe asthma patients with CKD risk factors and elevated inflammatory markers. The bidirectional relationship between asthma and DM is well-established. Clinicians should be mindful of asthma development in DM patients and vice versa, particularly in severe asthma cases receiving high doses of ICS or OCS. An increase in systemic inflammation markers and/or a decline in lung function in asthma patient with DM should prompt further assessment and more aggressive treatment. Asthma poses a risk for unfavorable lung cancer outcomes. Although the current evidence does not definitively establish lung cancer as a risk for asthma outcomes, optimal management of asthma in lung cancer patients with asthma is crucial to reducing the risk of poor outcomes in lung cancer. Obesity is identified as a risk factor for poor asthma outcomes. Asthma patients with obesity should undergo personalized management therapy early on, employing optimal treatment to achieve well-controlled asthma. Regular evaluation of lung function in cases of asthma-obesity that have not achieved well-controlled asthma also merits attention. Asthma is a risk factor for developing CVD. Patients with prolonged or severe asthma at risk for CVD should undergo a CVD risk stratification assessment and, if necessary, appropriate examinations. Managing asthma in older age requires careful diagnosis to differentiate between asthma, ACO, or COPD to ensure patients receive the correct treatment. In the case of occupational lung disease, it is crucial to address occupational exposure and comorbidities in workers with occupational asthma to enhance overall outcomes. The assessment and approach to chronic comorbidities are crucial in asthma management to achieve favorable asthma outcomes.

## Author contributions

AL: Conceptualization, Writing – original draft, Writing – review & editing. RO: Writing – review & editing. TH: Writing – review & editing. GI: Writing – review & editing. AY: Conceptualization, Supervision, Writing – review & editing.
